# Antimicrobial and Cytotoxic Effects of Cannabinoids: An Updated Review with Future Perspectives and Current Challenges

**DOI:** 10.3390/ph15101228

**Published:** 2022-10-06

**Authors:** Mansab Ali Saleemi, Noorfatimah Yahaya, Nur Nadhirah Mohamad Zain, Muggundha Raoov, Yoke Keong Yong, Nurul Shahfiza Noor, Vuanghao Lim

**Affiliations:** 1 Advanced Medical and Dental Institute, Universiti Sains Malaysia, Bertam, Kepala Batas 13200, Penang, Malaysia; 2 Department of Chemistry, Faculty of Science, Universiti Malaya, Kuala Lumpur 50603, Selangor, Malaysia; 3 Department of Human Anatomy, Faculty of Medicine and Health Sciences, Universiti Putra Malaya, Serdang 43400, Selangor, Malaysia

**Keywords:** *Cannabis sativa*, phytocannabinoids, structure–activity relationships, mechanism of action, cytotoxic effects

## Abstract

The development of new antibiotics is urgently needed to combat the threat of bacterial resistance. New classes of compounds that have novel properties are urgently needed for the development of effective antimicrobial agents. The extract of *Cannabis sativa* L. has been used to treat multiple ailments since ancient times. Its bioactivity is largely attributed to the cannabinoids found in its plant. Researchers are currently searching for new anti-infective agents that can treat various infections. Although its phytocannabinoid ingredients have a wide range of medical benefits beyond the treatment of infections, they are primarily associated to psychotropic effects. Different cannabinoids have been demonstrated to be helpful against harmful bacteria, including Gram-positive bacteria. Moreover, combination therapy involving the use of different antibiotics has shown synergism and broad-spectrum activity. The purpose of this review is to gather current data on the actions of *Cannabis sativa* (*C. sativa*) extracts and its primary constituents such as terpenes and cannabinoids towards pathogens in order to determine their antimicrobial properties and cytotoxic effects together with current challenges and future perspectives in biomedical application.

## 1. Introduction

Antibiotics have saved countless lives from infections around the world since Alexander Fleming discovered penicillin in 1928. The inappropriate use of antibiotics has led to the development of resistance to antibiotics. The overuse of antibiotics is a major contributor to the development of antibiotic resistance, which is threatening global health. The increasing number of multi-drug resistant organisms (MDRs) is a result of insufficient efforts in resolving the issue of antimicrobial resistance [1]. Due to the emergence of MDR pathogens, the availability of antibiotics for treating these infections is becoming increasingly limited. Alternative approaches are being studied and helper molecules are attracting attention, such as resistant breakers or antibiotic potentiators [2]. Helper molecules are non-antibiotic molecules that work synergistically as adjuvants for antibiotics through different mechanisms involving variations in membrane permeability, enzyme inhibition, and the inhibition of efflux pumps, all of which can participate in increasing specific antibiotic efficacies [3,4]. Some drugs that contain helper molecule characteristics are not only generally used to treating infectious diseases but also contain antimicrobial activities of their own [5]. A class of molecules known as helpers is known to block the activity of a membrane transport protein in the central nervous system. They are also linked to the side-of-action of certain drugs in the brain [5]. The inappropriate use of drugs is the main cause of microbial resistance. The World Health Organisation (WHO) reported that MDR is one of the biggest threats to the global healthcare system. For patients with certain immune-compromised conditions, such as those receiving chemotherapy treatment, being vulnerable to infection is often the case [6,7]. The development of new medical procedures and techniques is threatened by the emergence of multi-drug-resistant organisms. Therefore, the development of new class of antimicrobial agents seems to be necessary to tackle these problems associated with the healthcare system. For this, it is necessary to combine antibiotics with helper molecules in order to inhibit microbial growth. This approach may reduce the possibility of microbial resistance development and evaluations to detect effective helper molecules are, thus, crucial.

The herbaceous species known as *Cannabis sativa* L. is a member of the Cannabinaceae family [8]. Humans have been using these plants for thousands of years for various purposes, such as medicinal and recreation, initially in Northeast and Central Asia and gradually spreading globally [9]. The psychoactive substance known as marijuana or cannabis has a complex chemical composition that includes multiple cannabinoids [10]. A class of secondary metabolites known as cannabinoids has psychoactive effects [11,12]. Cannabinoids are divided into two categories: endogenous cannabinoids, which are created by the human body, and exogenous cannabinoids, which can be produced synthetically or by the *C. sativa* plant. The two G-protein coupled receptors that make up cannabinoids are known as CB1 and CB2. These receptors are part of the endocannabinoid system in human body [13]. Both exogenous and endogenous cannabinoids are receptor ligands, with tetrahydrocannabinol (THC) serving as the best exogenous ligand. Moreover, it not only serves as an agonist for CB1 and CB2 receptor-mediating effects, for instance, antiemetic effects, analgesia and muscle relaxation, but also contributes to harmful effects such as sedation, anxiety, and psychosis. However, cannabidiol (CBD) is another exogenous cannabinoid that has been found to minimise THC’s adverse side effects. It acts as antagonist for CB1 and CB2 receptors, causing anxiolytic, anti-sedative, and anti-psychotic effects [14]. In addition, it has the potential to cause many other effects such as anti-inflammatory effects [15] and prevents cancer cell growth [16] and the neuroprotection of neuro-degenerative diseases such as post-ischemia and Parkinson’s syndrome [17]. In addition, the use of cannabis has some adverse effects, which preclude its widespread use as a medicinal agent. For example, cannabis is well-known to cause psychological effects, such as euphoria, impaired motor skills, and the intensification of sensory perceptions in healthy individuals [18]. It has also been associated with deficits in episodic memory, anxiety, and executive functions [19,20,21]. Currently, a study conducted by the WHO proposed a relationship between stroke or myocardial infarction with cannabis use [22]. The activation of the CB1 receptor is known to cause adverse effects in the cardiovascular system; however, the effects of CBD are beneficial [23].

To date, very little information is available on the antimicrobial effects of cannabinoids and their mechanism of action. More research is required on their antimicrobial activity in order to understand the types of interaction between cannabinoids and pathogens. In this review, we provide a concise overview on the structure–activity relationship of cannabinoids and their early and recent antimicrobial activity or mechanism in order to review and discuss the adverse effects of cannabinoids.

## 2. Structures and Origin of Natural Cannabinoids from *Cannabis sativa*

**Figure 1:**
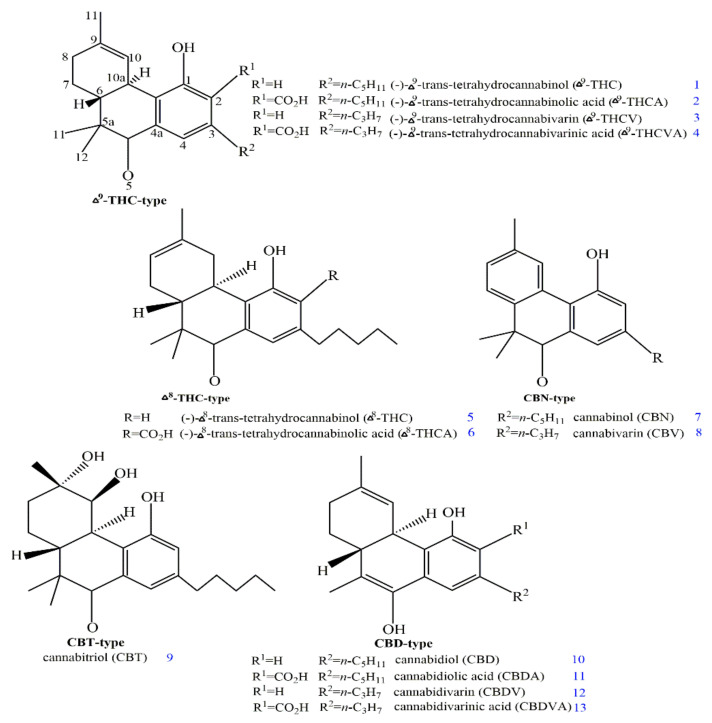

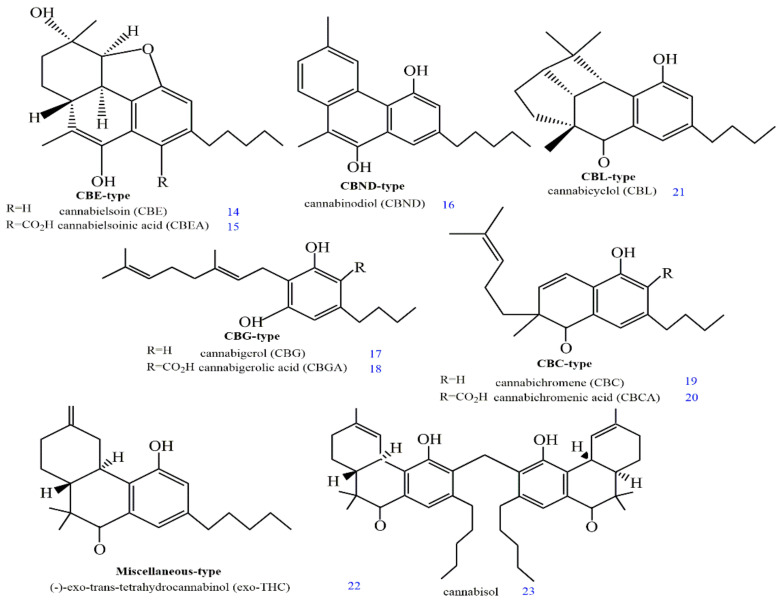
The structures of various phytocannabinoids derived from *C. sativa* (created with ChemDraw Professional).

*C. sativa* naturally produces cannabinoids, which typically have C21 and C22 terpenophenolic structures with varying oxidation patterns. There are currently approximately 120 phytocannabinoids discovered, and they can be divided into 11 different broad skeletal types, as summarised in Figure 1 [9,24]. The type of cannabinoids, such as delta-9-tetrahydrocannabinol (Δ^9^-THC-type) (1) [25], contains a tricyclic 6a,7,8,10a-tetrahydro-6H-benzo[c]chromen-1-o1 core structure, and its main representatives such as (-)-Δ^9^-trans-tetrahydrocannabinolic acid (Δ^9^-THCA) (2) and (-)-Δ^9^-trans-tetrahydrocannabinol (Δ^9^-THC) (1) include highly abundant cannabinoids of *C. sativa* [25,26]. Moreover, a class of cannabinoids such as Δ^8^-THC-type contains isomers of class Δ^9^-THC-type, demonstrating the same 6a,7,8,10a-tetrahydro-6H-benzo[c]chromen-1-o1 core structure with a double bond [9]. The Δ^8^-trans-tetrahydrocannabinol (Δ^8^-THC) (5) is considered to be a major representative of this form and its concentration in plants is usually negligible due to the isomerisation of thermodynamically less stable double bond isomers such as Δ^9^-THC (1) [25]. Cannabinoids of the cannabinol (CBN)-type share a similar core structure of 6H-benzo[c]chromen-1-ol with oxidised aromatic rings [9]. A comparatively minor constituent of *C. sativa* such as cannabinol (CBN) (7) is the primary representative of this class [25]. Although the content of CBN (7) increases in plant materials when oxidised, Δ^9^-THC (1) is processed in the presence of oxygen [27]. High concentrations of the thermodynamically more stable cannabinoids CBN (7) and Δ^8^-THC (5) can be found in processed cannabis products such as hashish and cannabis oil [27]. Additionally, the family of CBT-type cannabinoids, including cannabitriol (CBT) (9), clearly distinguishes itself from the Δ^9^-THC-type cannabinoids by exhibiting a vicinal 9,10-trans-diol in the upper ring [9].

However, CBD-type cannabinoids show a tetrahydro-[1,1-biphenyl]-2,6-diol framework with large amount in *C. sativa* and can synthesise its dried extracts up to 40% [9,28]. CBD (10) is inherently instable and cyclises to Δ^9^-THC (1) under acidic conditions [29]. The cyclisation process and oxidation of Δ^9^-THC (1) to CBN (7) also occurs during pyrolysis [30]. The oxidative photocyclisation of CBD-type cannabinoids produced CBE-type cannabinoids, such as cannabielsoin (CBE) (14), that have a 5a,6,7,8,9,9a-hexahydrodibenzo[b,d]furan-1,6-diol framework [9,31]. The CBG-type cannabinoids, for instance, cannabigerol (CBG) (17), demonstrate a non-cyclised framework that are minor constituents in *C. sativa* that normally convert into Δ^9^-THC-type cannabinoids during plant growth [9,32]. Moreover, cannabichromene (CBC) (19) is the most abundant CBC-type cannabinoids found in *C. sativa* [33]. The CBC (19) exposure to sunlight causes a [2 + 2]-photocycloaddition, forming cannabicyclol (CBL) (21) [34]. *C. sativa* can be split into a variety of other miscellaneous cannabinoids, such as dimeric cannabisol (23) or (-)-exo-*trans*-tetrahydrocannabinol (exo-THC) (22). Phytocannabinoids present a C22 and C19 terpenophenolic structure, including (-)-Δ^9^-trans-tetrahydrocannabivarin (Δ^9^–THCV) (3) or cannabidivarin (CBDV) (12), as shown in (Figure 1) [35,36,37]. The terpenophenolic structure originates from the olivetolic acid (28) and monoterpene precursor geranyl diphosphate (GPP) that is synthesised via the olivetolic acid cyclase and a polyketide synthetase (PKS) [38,39]. The cannabigerolic acid synthetase (CBGAS) facilitates the catalysis of prenyl transfer by an electrophilic aromatic substitution, leading to the production of cannabigerolic acid (CBGA) (18) [40].

In contrary, the production of cannabidiolic acid (CBDA) (11) through an oxidation cyclisation is catalysed by cannabidiolic acid synthetase (CBDAS) [41]. Cannabichromenic acid (CBCA) (20) is also produced through oxidation cyclisation (13) and catalysed by the cannabichromenic acid synthetase (CBCAS) [42], although decarboxylation reactions also provide contributions to some extent during the smoking or baking of cannabis materials [43].

## 3. Structure–Activity Associations of Cannabinoids

It is widely known that cannabinoids have antimicrobial effects. Despite the various advantages of cannabinoids, their potential in helping combat antibiotic resistance is still largely untapped. Some studies have been conducted on the subject, as listed in Table 1.

The researchers observed that both cannabinoids were active towards various types of bacteria, including those that are known to cause respiratory infections (*S. aureus* and *Streptococcus* species). Their results indicated that the compounds had an MIC of 1–5 µg/mL [44]. Although the binding of plasma proteins to the cannabinoids in the horse serum reduced its antibacterial activity against pathogenic bacteria, it did not impair the serum’s ability to kill germs. In order to determine the properties of cannabichromene analogs, researchers tested the antimicrobial and anti-antibiotic properties of these substances [45]. The N-pentyl chain meta relative to the alcohol group is known to play a role in the development of antibiotic resistance against two bacterial species, namely *B. subtilis* and *S. aureus*. However, truncation to a methyl group is associated with an increase in antifungal activity. Moreover, the activity of isocannabichromenes was studied, but it was not as active as their analogs cannabichromene. This class of cannabinoids does not cause psychoactive effects, but it can improve its therapeutic potential. In one study, authors studied the performance of various cannabinoids on the development and maintenance of antibiotic resistance against multidrug-resistant strains of *S. aureus* [46]. A study conducted on cannabidiolic acid revealed that it has good antimicrobial activity (MIC = 2 µg/mL).

It was also found that the presence of a carboxylate moiety did not affect its activity. According to the researchers, when it comes to treating various Gram-positive pathogens, CBD (10) has a MIC value of up to 1–2 µg/mL, which is significantly higher than CBDA (11) [47]. The inactive compounds in CBDA (11) were phenethyl and methyl. These could have been induced by the added hydrophobicity or by the steric bulk. The effects of acetylation and methylation on various hydroxyl groups were detrimental to the activity of microbial cells. The removal of the carboxylate increased the moderate activity of CBGA (18). Compared to CBND (16) and ∆^9^-THC (1), ∆^9^-THC acid exhibited a moderate bactericidal effect. Interestingly, the effects of switching to the N-pentyl group from the hydroxyl group were not significantly affect the antimicrobial activity. A study conducted on resorcinol not only revealed that it exhibited poor antimicrobial properties but also showed the importance of the hydrocarbons chain.

Currently, researchers evaluated the effects of endocannabinoid anandamide and arachidonyl serine on bacteria [48]. Despite their poor bactericidal activity against certain types of bacteria such as methicillin resistant *S. aureus* (MRSA), these compounds inhibited the formation of bacterial biofilms [48]. The changes induced by these compounds affected the cell aggregation, hydrophobicity, and membrane potential of various bacterial species. When combined with other antibiotics such as ampicillin, these agents can be used to treat MRSA-caused infections that recur [49]. It has been demonstrated that CBD (10) can improve the antibacterial effects of the peptide drug bacitracin against many bacteria, including *L. monocytogenes* and *E. faecalis* [50].

In another study, researchers evaluated various cannabinoid analogs against *E. coli* and MRSA [51]. Several common cannabinoids exhibited moderate to good activity when used in combination with other drugs. The increase in the minimum inhibitory concentration (MIC) values of various analogs, such as ∆^9^-tetrahydrocannabivarin, due to the presence of a common n-propyl chain, which further highlighted the importance of this component in the membrane insertion process. Hydroxylation and carboxylation at position 11 of the ∆^9^-THC (1) resulted in a loss of activity, which suggests that the presence of a lipophilicity in the prenyl tail may be important. CBG (17) was able to reduce the bacterial burden in the spleen in a mouse model of a systemic infection with MRSA by a factor of 2.8 log^10^ in colony-forming units. Although these analogs did not exhibit a bactericidal effect against *E. coli*, their consistent MIC values were over 128 µg/mL. In a study, CBG (17) was shown to be effective against Gram-negative bacteria by combining with polymyxin B. It is proposed that the polymyxins be added to the outer membrane of a Gram-negative pathogen to enable the CBG (17) to perform its functions. The study also revealed that cannabidiol can sensitise various antibiotics in combination with other drugs [52]. For various Gram-negative bacteria, CBD (10) was able to prevent the release of membrane-filled cargo containers. These containers play a vital role in inter-bacterial communication. When combined with other antibiotics, such as vancomycin, colistin, and erythromycin, CBD (10) was able to enhance the antimicrobial effect towards *E. coli*. The results of previous studies suggest that cannabinoids can potentially improve the efficacy of existing antibiotics.

**Table 1:** The activities of *C. sativa* and cannabinoids against the pathogens enlisted in World Health Organisation’s latest priority list.

Bacterial Strains	Compound/Extract/Essential Oils	Activity	Reference Antibiotic	Outcomes	Ref.
*P. aeruginosa*	Aqueous extract	MIC 7.14 mg/mL	Ciprofloxacin	A higher anti-inflammatory and antioxidant profile was shown by the water extract, along with a significant inhibition on the selected pathogen.	[53]
	Plant extract	MIC 12.5 µg/mL	-	The plant extracts show considerable antibacterial activities against *P. aeruginosa*.	[54]
*N. gonorrhoeae*	CBD (10)	MIC 1–2 µg/mLMIC 0.03–16.0 µg/mL	Vancomycin, Levofloxacin, Meropenem, Gentamicin, Mupirocin, Colistin	The findings show that cannabidiol has superior anti-biofilm activities, limited tendency to cause resistance, and topical in vivo efficacy. Various investigations on the mechanisms of action of cannabidiol point to membrane disruption as the main mechanism.	[55]
*Staph aureus*, *Lactobacillus*	Seed extract	MIC 2.5 mg/mL	-	The results of the study revealed that *C. sativa* extracts can effectively treat pathogenic strains. It also did not affect the growth of beneficial bacteria.	[56]
*P. aeruginosa*, *E. coli*	Essential oil	MIC 1.2 mg/mL	-	The use of *C. sativa* essential oil as a potential source of antimicrobials and natural antioxidants could offer a promising strategy to treat various infectious diseases.	[57]
*E. coli*, *Salmonella typhimurium*	Seed extract	Growth inhibition at MIC 1 mg/mL	-	It has been observed that *C. sativa* extracts had selective antimicrobial action against pathogenic strains and had no negative effects on the growth of probiotic strains.	[56]
*E. coli*	Seed extract	MIC 25 µg/mL	-	The plant extracts show higher antibacterial activities against pathogens.	[54]
	N-*p*-trans-coumaroyl-tyramine	IC_50_ 0.8 µg/mL	Ciprofloxacin	The compound displayed strong antibacterial activities against bacteria.	[58]
	Aqueous extract	MIC 7.14 mg/mL	Ciprofloxacin	A higher anti-inflammatory and antioxidant profile was shown by the water extract, along with a significant inhibition on the selected pathogen.	[53]
Vancomycin-resistant Enterococci	CBCA (20)	MIC 7.8 µM	-	It was observed that CBCA (20) demonstrated faster and more potent bactericidal activity than vancomycin. Microscopical analysis reveals that CBCA (20) may work by altering the bacterial nucleoid and degrading the lipid membrane of the bacterial cell.	[59]
*S. pneumoniae*	CBD (10)	MIC 1–4 µg/mL	Vancomycin, Daptomycin, Trimethoprim, Mupirocin, Clindamycin	The findings show that cannabidiol has superior anti-biofilm activity, limited tendency to cause resistance, and topical in vivo efficiency.	[55]
MRSA, *E. faecium*	CBD (10)	MIC 1–2 µg/mL	Vancomycin, Daptomycin, Trimethoprim, Mupirocin, Clindamycin	Various investigations on the mechanisms of action of cannabidiol point to membrane disruption as the main mechanism. Moreover, cannabidiol has superior anti-biofilm activity, limited tendency to cause resistance, and topical in vivo efficacy.	[55]
EMRSA 15, EMRSA 16	CBD (10), ∆^1 & 9^-THC (1), CBG (17), CBC (19), CBND (16)	MIC 0.5–2.0 µg/mL	-	The compounds demonstrated strong antimicrobial activity against various MRSA strains with contemporary clinical significance.	[46]
	CBD (10), ∆^1 & 9^-THC (1), CBG (17), CBC (19), CBND (16)	MIC 1–4 µg/mL	Ciprofloxacin	The results of the study showed that five of the hemp essential oils inhibited the growth of pathogens. This suggests that these can help reduce bacterial populations in the environment.	[60]
*E. faecium*	Essential oil, α-humulene, α-pinene, β-pinene, myrcene	MIC 0.75–1.87 (%, *v/v*) MBC 1.39–2.83 (%. *v/v*)	-	Essential oils extracted from industrial hemp can help prevent the growth of harmful microbes. This benefit can be achieved depending on the variety and sowing time.	[61]
	Essential oil	IC_50_ 0.82–4.22 µg/mL	-	The essential oil showed potent and selective antibacterial activity against selected bacteria.	[62]
	CBG (17)	MIC 2 µg/mL and MBEC 4 µg/mL	-	The study shows that the drug can target the membrane of Gram-positive bacteria. It also shows that the drug can be effective in treating an infection caused by MRSA in a mouse model.	[51]
	CBDA (11)	MIC 4 µg/mL	Tobramycin, Meropenem, Ofloxacin	The compound had strong antibacterial activities towards bacterial strains and may be used as a substitute drug to treat MRSA.	[47]
MRSA	CBD (10), CBND (16), CBC (19), CBDV (12) and ∆^1 & 9^-THC (1)	IC_50_ 5.8–10.6 µM	Ciprofloxacin	All compounds showed antimicrobial properties when tested for antibacterial activity against a panel of pathogens.	[63]
	CBD analogs	MIC 0.25–64.0 µg/mL	Vancomycin, Daptomycin, Mupirocin	The findings show that cannabidiol has superior anti-biofilm activity, limited tendency to cause resistance, and topical in vivo efficacy.	[55]
	CBD (10)	MIC 1 µg/mL	Tobramycin, Meropenem, Ofloxacin	CBD (10) had a potent antibacterial activity against Gram-positive strains and may be used as a substitute drug to treat MRSA.	[47]
	CBCA (20)	MIC 3.9 µM	-	Microscopical analysis reveals that CBCA (20) may work by altering the bacterial nucleoid and degrading the lipid membrane of the bacterial cell.	[59]
	4-acetoxy-2-geranyl-5-hydroxy-3- n-pentylphenol	IC_50_ 6.7 µM	Ciprofloxacin	Compounds displayed significant antibacterial activities towards MRSA.	[64]

## 4. Antimicrobial Activity of *Cannabis sativa*

The report about the antibacterial properties of cannabinoids was first published in the 1950s [65,66]. The bactericidal properties of cannabis were studied before the phytochemistry of the plant was fully established. This means that the antibacterial effects of *C. sativa* were not attributed to a specific component. In 1976, it was discovered that ∆^9^-THC and CBD (10) can be used as bacteriostatic agents. They were also able to kill a panel of human pathogenic strains [44]. The antibacterial properties of the various *C. sativa* plant extracts have drawn significant attention, such as the oil and extract from the plant. Various methods have been used to isolate *C. sativa* extracts. Cold-pressing and solvent extraction techniques are commonly used to produce various products, such as cosmetics and food. However, new technologies are now being developed that allow them to generate superior results [67]. Pressurised liquid extractions are more efficient than filtration. They do not require filtration and have shorter processing times. On the other hand, ultra-sonic extraction techniques use less solvent and have improved yields. There are various methods that are commonly used for green extraction, such as supercritical fluid extraction and microwave-assisted extraction; however, up-scaling these processes is challenging [67].

Essential oils from five different cultivars of *C. sativa* were evaluated against a panel of Gram-negative and Gram-negative pathogens. The most common compounds found in oil samples were trans-β-ocimene, myrcene, and trans-caryophyllene, but they showed less antibacterial activities against *Brevibacterium linens* and *Acinetobacter calcoaceticus*. A comprehensive analysis of the various essential oils revealed that none of them had high levels of ∆^9^-THC (1) and CBND (16). These compounds, which are known to be antimicrobials, could be utilised by *C. sativa* [68]. A study was conducted on the oil of the seeds, which were then extracted using petroleum and methanol. The agar diffusion method was used to extract antimicrobial properties from various extracts. It was shown that the extract exhibited effective responses towards different pathogenic strains. The lack of a comprehensive analysis of the plant’s cannabinoid content is consistent with the findings of the study conducted by researchers [44]. There was no obvious antifungal activity observed. A small amount of petroleum ether extract was also observed to have beneficial effects against bacteria [54]. Inhibiting the development of harmful Gram-negative pathogens is also possible with the use of hot water and ethanol leaf extracts [8]. A study conducted on *C. sativa* shows that the plant’s antioxidant and antimicrobial properties were compared after both aqueous and acetone extraction [69]. Compared to aqueous extracts, acetone extracts exhibited superior bactericidal properties. The effects of varying concentrations on the responses of different bacteria were studied. The most responsive species was the *V. cholera* bacterium, closely followed by the *P. aeruginosa*. The study revealed that *C. sativa* has antioxidant properties, which could be useful in treating various conditions. A study conducted by researchers revealed that the drug “Hashish” can kill harmful bacteria [70]. The results of the experiments revealed that cannabis extracts significantly inhibited the growth of *S. aureus* 25923. The results of the study support the idea that the antimicrobial properties of *C. sativa* plants grown in Vietnam are modest against Gram-positive bacteria. On the other hand, the extracts from cultivated strains of the plant exhibited less resistance to Gram-negative organisms [71]. The researchers also noted that the major components of the extracts exhibited moderate activity against Gram-positive pathogens [72].

Due to their low toxicity, hemp seed oil-based products are investigated for cosmetic and pharmaceutical applications. The antimicrobial properties of two different types of oil-based emulsions were determined. For instance, the activity of oil-based emulsions against *E. coli* was virtually zero. This might be a result of the higher concentration of α-linolenic acid or, more likely, the removal of ∆^9^-THC (1) during the refinement process [73]. The extract of *C. sativa* has been studied with respect to various types of antibiotic-resistant bacteria, such as MRSA, by using the disc diffusion method. The zone of inhibition of clinical isolates was observed as 9 to 15 mm. This was less than the diameter of vancomycin (13–24 mm). A combination of plant extracts, such as *Psidium guajava* and *Thuja orientalis*, exhibited a synergistic effect. Zone of inhibition diameters of up to 30 mm were observed in most cases. Flavonoids, such as quercetin, catechin, and gallic acid, were found in the leaf extract, but no traces of cannabinoids were detected [74]. In vitro studies conducted by scientists revealed that the extract of *C. sativa* inhibited the formation of *S. aureus* biofilms [56]. In a study conducted on dental plaque, researchers found that using cannabinoids can help decrease the bacterial colony count in the plaque. They also compared the effectiveness of these products with those from commercial brands such as Colgate [75].

Investigators are currently examining the commercial viability of ∆^9^-THC-free essential oils from *C. sativa*, which could be used for various applications such as veterinary medicine and cosmetic products. The oil was evaluated against various strains of *S. aureus*, and it exhibited moderate antibiofilm activities and antibacterial effects. Moreover, antimicrobial activities were detected against *Helicobacter pylori* but not against other organisms. The study shows that the active compounds found in *C. sativa* are not only capable of acting as antimicrobial agents, but they also have biological properties [76]. A wide range of applications for hemp-seed hexane extracts has been studied, which exhibited that the oil extracted from this plant can also help in reducing acne-causing bacteria and can help prevent inflammation [77]. In order to better understand the properties of various essential oils, researchers conducted a comprehensive phytochemical analysis of 17 different types of hemp essential oil. A total of 71 compounds were identified, while some of these include terpene β-myrcene, trans-ocimene, and limonene. The inhibitory concentration of various oils was analysed against a group of Gram-positive bacteria, and they were able to show moderate antimicrobial activity. The effects of various cannabinoids, such as CBD (10) and terpene, on the development of the antibacterial effects were studied. Although activity was generally good to moderate against various types of bacteria, such as *Enterococcus* and *Salmonella*, it was lower against other types of bacteria. The antimicrobial effect of essential oils is likely caused by the presence of synergism between different compounds [60]. An interesting use for compounds derived from *C. sativa* is in water purification in order to isolate a combination of compounds, including terpene and cannabinoids; they immobilised them on a polyethersulfone hybrid membrane. The reduction in bacterial populations was observed for both Gram-negative and Gram-positive pathogens. Several different bacterial species, including common pathogens *E. coli* and *P. aeruginosa*, were found to have similar results. This study aims to provide a cost-effective solution for the treatment of waterborne pathogens by using a combination of water filtration and purification [78]. The great potential of *C. sativa* for various applications in drug discovery is highlighted by its antimicrobial properties.

## 5. Antibacterial Mechanism of Action

**Figure 2:**
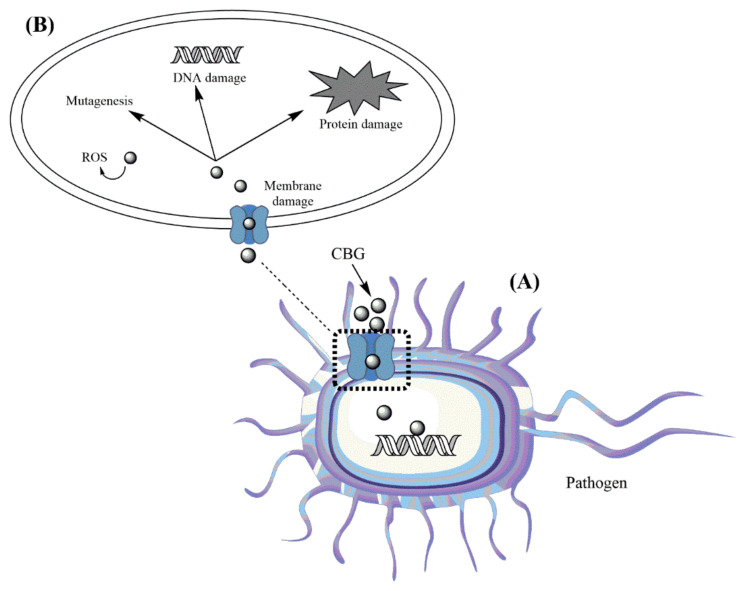
Schematic illustration of antibacterial mechanism of cannabinoid, with CBG (17) as an example. CBG (17) causes bacterial cytoplasmic membrane damage (**A**) that results in the destruction of protein and DNA and the production of reactive oxygen species (ROS) within the bacterial cell (**B**) (created with ChemDraw Professional).

Despite the lack of an effective mechanism of action for treating bacterial infections, recent advances have been made in the field of cannabinoids. Membrane permeability is one of the cannabis compounds’ potential mechanisms of action. *L. monocytogenes’* cell integrity and wall structure were both disrupted by the terpene limonene, which caused a leakage of several cell components [79]. Similar changes to those caused by β-caryophyllene were observed in the *Bacillus cereus* bacterium’s membrane [80]. CBG (17) has shown that it can target the cytoplasmic membrane of Gram-positive bacteria, as shown in Figure 2. Gram-negative bacteria’s inner membrane was permeabilised, enabling CBG (17) to perform in a manner comparable to that of Gram-positive bacteria [51]. A microscopic assessment of the efficiency of CBCA (20) on the development of *B. subtilis* showed that it induced a change in the bacterial membrane and nucleoid [59]. In vitro studies revealed that CBD (10) caused a depolarisation of the membrane of *S. aureus*, while this activity also disrupted the membrane potential of the bacterium. The combination of CBD (10) and bacitracin can cause various cell division defects and cell envelope abnormalities. It is believed that the abnormalities were caused by a loss of genes that regulates the division of cells [81].

Another mode of action of cannabinoids that can be used to alter cell communication is by blocking the release of membrane vesicles by bacteria. Although it was shown that CBD (10) can block the release of membrane vesicles from the pathogen, this effect was not significant in the presence of *S. aureus* [52]. Moreover, the effects of HU-210 on the bacterial communication system were studied, which showed that the drug can inhibit the quorum sensing (QS) system’s ability to detect and respond to bacterial signals. It was also able to improve the swimming performance of *Vibrio harveyi* [82]. In one study, a radiolabeled synthesis test in *S. aureus* RN42200 revealed that various pathways that led to the synthesis of proteins, DNAs, and RNAs were significantly inhibited by concentrations near an MIC of 2–3 µg/mL^−1^ [55]. This suggests that rapid bactericidal action is carried out to shut down these pathways [83]. The reduction in lipid synthesis was observed at concentrations below the MIC, which supports the hypothesis that membrane-based effects were involved [46]. The presence of a membrane depolarisation in the presence of MRSA can provide additional evidence of membrane activity; however, this activity was not observed in *E. coli* [55]. A bacterial cytological profiling test performed on multiple antibiotics known to act through membrane permeabilisation showed that the results were consistent with previously published results [84,85]. The results of these studies suggest that CBD (10) can be very effective at disrupting bacterial membranes; however, it is not clear if this effect is caused by a specific molecular target.

## 6. Heat Map Clusters of Cannabinoids

**Figure 3:**
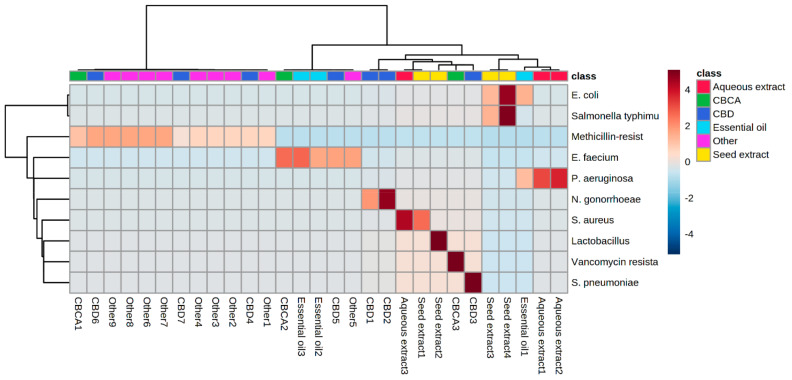
Heat map clusters of different compounds of cannabinoid, illustrating the correlation between samples and different microbial species. The structure produced from MetaboAnalyst from https://www.metaboanalyst.ca/ (accessed on 13 July 2022).

The quantitative values of various compounds of cannabinoids were compared with those of microbial strains. The correlation between these values and the strains of microbes was also discussed, as shown in Figure 3. It was observed that CBD 2 and 3 (10) show a positive correlation against *N. gonorrhoeae* and *S. pneumoniae* due to the structure–activity relationships of cannabidiol analogs, while CBD 1 (10) has a negative correlation towards bacterial strains. Various factors that affect the development and maintenance of microbial strains in a variety of cannabidiol analogs can be considered. Some of these include the size, concentration, and exposure time of cannabinoids [55]. Different strains of bacteria were used in different studies, which means that the results of the studies were different from those of the previous studies. Moreover, CBCA 3 (20) exhibits the most significant antimicrobial property due to its strong interactions with the microbial cell membrane, the induction of oxidative stress inside the cell, and the disruption of the metabolic function, resulting in microbial cell lysis. Both concentration and exposure time play a very important role in improving the antimicrobial activity of different cannabidiol analogs. Previous studies show that antimicrobial activity increases as the concentration and exposure time increase [55]. Similarly, seed extract 2 shows a positive correlation against bacterial cells due to a number of proteins and peptides produced by seed extracts with antibacterial activities, while seed extract 1 demonstrates a less significant correlation towards bacterial strains. Aqueous extract 3 also demonstrated significant antibacterial activities against *S. aureus* due to strong bacterial cell membrane interaction. However, various others compound of cannabinoids demonstrate an intermediate level of correlation against different bacterial strains because of weak electrostatic interactions between compounds and microbes. With the potential to create novel analogs with narrow spectrum, selective Gram-negative activity against the harmful pathogen *N. gonorrhoeae*, CBD (10) represents the prototype member of a promising structural class of antibiotics [55]. A new class of compounds has been discovered that is capable of treating the most common forms of gonorrhoeae resistance. However, it is not yet clear if these compounds can be utilised in a systemic manner due to concerns about the emergence of “superbugs” [67]. Trials are currently being conducted with respect to the use of CBD (10) in treatments for other conditions, such as nasal colonisation [55]. Studies have shown that cannabidiol compounds have a potential to become a useful new antimicrobial agent.

## 7. Cytotoxic Effects of Cannabinoids

**Figure 4:**
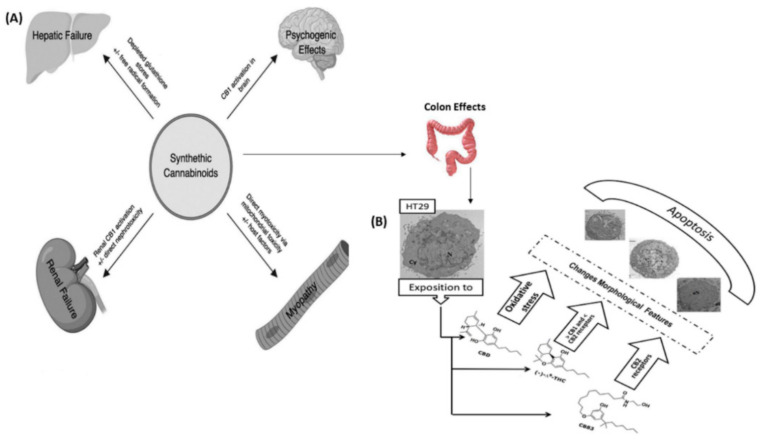
Schematic illustration of (**A**) proposed mechanisms of synthetic cannabinoids end-organs effects, and (**B**) their cytotoxic effects on human HT-29 colorectal adenocarcinoma cells (reprinted with permission from [120,121]; copyright (2019) (2020) ACCP; MDPI; the articles were printed under a CC-BY license).

Cannabidiol is a major phytocannabinoid found in cannabis plants. It is regarded as one of the most extensively studied compounds in the plant. CBD (10) interacts with a wide variety of physiological targets in one’s endocannabinoid system. CBD (10) is frequently prepared as an oil and is most frequently taken orally in medical settings. Unlike other psychoactive substances, such as THC, cannabidiol is non-intoxicating, which makes it an appealing treatment for various conditions. In general, synthetic cannabinoid toxicity is a major cause of organ injury in patients, as shown in Figure 4A. Synthetic cannabinoid toxicity can be identified by the presence of certain clinical characteristics, such as a high index of suspicion. This helps identify the most effective organ-specific interventions for improving patient outcomes. There are currently several studies looking into the potential of cannabidiol to treat cancer. Two recent studies on the effects of cannabidiol on cancer showed that it can reduce the risk of cancer [86,87]. In addition to these, studies also suggest that treatments with CBD (10) can help prevent cancer cells from growing in various other organs, such as the colon, breast, lung, prostate, cervical, brain, melanoma, neuroblastoma, leukemia, and multiple myeloma cancer cells [86,87]. Table 2 shows the latest studies on various cannabinoids used in cancer models [88].

### 7.1. Colon Cancer

In vitro studies revealed that cannabidiol significantly decreased the viability of colon cancer cells and elevated the levels of certain nutrients in the cell [115,116]. It also promoted the development of cancer cells’ apoptosis. CBD (10) significantly decreased the number of tumours and crypt foci in animal model. An inhibition of colon cancer cells by the upregulation of a protein known as caspase-3 was observed in CBD (10) [115]. Other in vitro studies show that treating colon cancer with CBD (10) can help decrease the cancer cells’ proliferation and induce apoptosis, as shown in Figure 4B. It also has anti-angiogenesis and anti-metastatic properties [117]. The antagonistic action of CBD (10) at GPR55 in HCT116 colon cancer cells was demonstrated to be a critical element in preventing and reducing metastasis [118]. The use of CBD (10) treatment resulted in a reduction in tumour volume and the production of specific pro- and anti-apoptotic proteins, according to in vitro colorectal cancer models [119].

### 7.2. Breast Cancer

Several studies have shown that CBD (10) can be beneficial in treating breast cancer. It has been shown that CBD (10) therapy encourages breast cancer cells to undergo apoptosis and autophagy [122,123]. It has been suggested that CBD (10) can cause endoplasmic reticulum-induced apoptosis via increasing the formation of ROS in breast cancer cells [123]. The study also revealed that CBD (10) can block the development of breast cancer cells by preventing them from using epidermal growth factor (EGF) [124]. CBD (10) is now being studied for its potential to prevent EMT in cancer cells. Treatment with CBD (10) has the potential to improve cell contact recovery, lessen the expression of specific cancer markers, and lessen breast cancer cells’ invasion and migration [124]. Through the use of CBD (10), it was also able to decrease the sensitivity of malignant phenotype (6D cells) to the multiple anticancer agents, such as cisplatin and doxorubicin [125]. CBD (10) decreased tumour size and growth in breast cancer-related mouse models, along with tumour migration and invasion to decrease metastasis [124]. The researchers noted that by blocking the activity of the gene, the treatment led to a significant decrease in the spread of cancer [126]. Another study found that CBD (10) therapy could lessen breast cancer cells’ ability to proliferate and invade by lowering Id-1’s helix–loop–helix protein expression [127], although Id-1 overexpression in breast cancer has been discovered to be closely related to the potential of primary human breast cancer cells with respect to spread to the lung [127]. Researchers studied the effects of CBD (10) on breast cancer cells after they were exposed to either doxorubicin or paclitaxel, and they observed that the combination of CBD (10) and these drugs could help decrease cancer cells’ damage. In addition to being effective as a monotherapy, CBD (10) nanoparticles can also prolong the antiproliferative activity of patients by up to 10 days. This suggests that they may help prolong the release of cannabinoids in patients [94].

### 7.3. Lung Cancer

It has been shown that CBD (10) can trigger the activation of the cyclooxygenase 2 and peroxisome proliferator-activated receptor gamma (PPARɣ) pathways to cause cell death of lung cancer cells [128]. Many studies have shown that CBD (10) can prevent the invasion and spread of lung cancer cells by decreasing the secretion of a plasminogen activator inhibitor-1 [129,130]. In addition, studies also suggest that CBD (10) can increase the activity of a protein known as the intercellular adhesion molecule (ICAM-1) in lung cancer cells, which is known to decrease the spread of disease. Furthermore, CBD (10) treatments rendered lung cancer cells more likely to attach to and be destroyed by lymphokine-activated killer (LAK) cells, and it was discovered that the overexpression of ICAM-1 was the cause of LAK cells’ enhanced activity [131]. In one study, CBD’s effects on lung cancer cell line proliferation, migration, and EMT were investigated. Researchers noted that treating lung cancer cells with CBD (10) resulted in a reduction in the cancer cells’ migration and a restoration of the cancer’s epithelial phenotype [132]. In vivo studies conducted on lung cancer mice revealed that treating them with 10 mg/kg/day of CBD (10) daily resulted in a reduction in their cell viability and decreased their overall tumour growth [128].

### 7.4. Prostate Cancer

Prostate cancer studies have also shown that CBD (10) has a number of interesting anti-cancer properties. CBD (10) therapy greatly reduced the growth of several prostate cancer cell lines [52,133]. In one study, CBD (10) suppressed the proliferation of prostate cancer cells by activating tumour protein p53, inducing intrinsic mechanisms of apoptosis, and arresting the cell cycle at the G1-S phase. In a mouse xenograft model, CBD (10) therapy was also effective at decreasing tumour development and enhancing the effects of docetaxel [133].

### 7.5. Neuroblastoma and Glioma

Cannabidiol has been demonstrated to have anti-cancer properties in gliomas. Treatment with CBD (10) reduced cell growth and triggered apoptosis in glioma cells [96,134]. A fascinating study found that CBD (10) reduced cell viability in a dose–dependent manner and that pure CBD (10) was superior to CBD (10) as a botanical therapeutic ingredient [135]. Researchers also observed that the enhanced reactive oxygen species (ROS) generation caused by CBD (10) led to the death of glioma progenitor cells [96]. Another study discovered that CBD (10) therapy enhanced the generation of ROS, which in turn caused an increase in the expression of heat shock proteins in glioma cells [136]. The cytotoxic effects of CBD (10) were retained in glioma cells when they were cultured with CBD and heat shock protein (HSP) inhibitors, even if increases in HSP rendered them less efficient [136]. Furthermore, glioma cells grown with CBD (10) and HSP inhibitors were more radiosensitive than those cultured with CBD (10) alone [136]. CBD (10) treatment was able to decrease tumour development, improve apoptosis, and considerably lengthen mouse survival in in vivo brain cancer models in mice [96,134]. Moreover, another in vivo study showed that temozolomide (TMZ) and both CBD (10) and Δ^9^-THC (1) were used in the treatment of glioblastoma multiforme (GBM). Researchers discovered that TMZ in combination with Δ^9^-THC (1) and CBD (10) in a 1:1 ratio and formulations richer in CBD (10), but not TMZ with CBD (10) alone, had equivalent anti-tumour effects in glioma cell-derived xenografts [95]. However, the combination of TMZ with cannabis preparations higher in CBD (10) demonstrated more potent anti-tumour effects made from glioma-initiating cells [95]. The same study examined the systemic administration of Sativex-like extracts (1:1, CBD (10):Δ^9^-THC (1)) in conjunction with TMZ and discovered that combination therapies could still have an anti-tumour effect [137]. In neuroblastoma cell lines, CBD (10) reduced invasion, cell proliferation, cell cycle arrest, and tumour development [128]. Another study shown that CBD (10) inhibited cell migration and invasion and promoted death in neuroblastoma cells by activating serotonin and vanilloid receptors [134]. Additionally, xenografted glioma-bearing mice were treated with CBD-loaded microparticles, which reduced tumour angiogenesis and cell proliferation [138].

### 7.6. Other Cancers

Cannabidiol has the ability to treat various types of cancer, such as leukemia, cervical, endometrial, and melanoma. Treatment with CBD (10) had benefits on mice given melanoma cells that were remarkably comparable to those of the anticancer drug cisplatin, such as boosting lifespan, significantly slowing the growth of the melanoma tumour, and raising the general quality of life [139]. A study conducted by researchers revealed that CBD (10) can decrease the viability of T lymphocytes and increase their numbers in the G1 phase of cell cycle in leukemia cells [110]. It was also known that CBD (10) exposure in leukemic cells triggered apoptosis caused by the accumulation of ceramide [140]. Scientists also revealed that CBD (10) decreased the expression of P-gp in certain cell types, such as CEM/VLB (100) cells. It also correlated with the accumulation of Rh123 and sensitised the cells to Vinblastine [141]. Cervical cancer cells were treated with CBD (10) at doses ranging from 1.5 µg/mL to 3.2 µg/mL, and this resulted in the inhibition of cell proliferation and death [142]. The resistance of malignancies to anti-cancer treatments is strongly correlated with ABC transporters. In ovarian cancer cells that were overexpressing ABCC1, CBD (10) treatments boosted the intracellular accumulation of two ABCC1 substrates, Vincristine and Fluo3 [143]. It was observed that CBD (10) increased the cytotoxicity of bortezomib and carfilzomib in multiple myeloma cells, decreased cell viability, and prevented cancer cells from migrating [144]. According to a different study, CBD (10) inhibited the multidrug transporter ABCG2 and facilitated the intracellular accumulation of the transporter’s substrate, mitoxantrone [143]. Concentrations of CBD (10) higher than 5 M significantly decreased cell viability in endometrial cancer. In Ishikawa cells, CBD (10) enhanced the levels of caspase 3/7, reactive oxygen species, and cleaved poly (ADP-ribose) polymerase (PARP), which suggests apoptosis induction. The activation of transient receptor potential cation channel subfamily V member 1 considerably aided CBD’s anti-cancer activity in endometrial cancer cells [145].

## 8. Current Challenges and Future Perspectives

The complexity of the legal boundaries surrounding cannabis has been recognised as a major factor that has hindered the development of effective CB research [146]. Due to the difficulty of complying with these regulations’ legal requirements, researchers and funding organisations may be less inclined to examine innovative products. It is anticipated that the research community will begin to invest in the development of new and improved methods for delivering CBDs as a result of the increasing acceptability of CBDs in the US and other nations. One of these methods includes developing transdermal and topical delivery systems. Therefore, the National Center for Complementary and Integrative Health has expressed interest in financing research into the study of CBDs [147]. Pharmaceutical companies or other research institutions may start a similar programme in the following years with an emphasis on the evaluation and development of topical or transdermal drug delivery system (TDDS) for CB usage, given the numerous benefits of this approach [148,149].

Due to the emergence of antibiotic resistance, there has been a search for new strategies and methods to treat bacterial infections. Many plant compounds and extracts have been demonstrated to possess antibacterial activity against a variety of pathogens. *C. sativa* compounds can be very attractive as they have various pharmacological properties. Although many compounds that have been studied in this area are already in their early stages, the potential of using Cannabis extracts as an effective antibiotic remains to be investigated. One study showed that the antimicrobial properties of both essential oils and cannabis extract can be found. The variation in the extracts analysed and the applied microbiological test is most likely the cause of the variable results in the spectrum of activity of cannabis products. It should be emphasised that the majority of the reports included in this evaluation were not thoroughly examined. Moreover, the compounds used in the production of cannabis extract were not fully assessed. Additionally, several conclusions have been made from the study of the various aspects of diffusion technique methods and their applications in the microbiological field. In terms of their active properties against Gram-positive bacteria, cannabis extracts and purified cannabinoids are more effective against multidrug-resistant organisms. Cannabinoids have been known to have antimicrobial properties against various bacteria, including those that are harmful to humans. In addition, cannabinoids can enhance the effectiveness of antibiotics by acting as a natural antimicrobial agent. Cannabinoids are excellent candidates for the development of new combination therapies because they can increase the efficiency of antibiotics against resistant microorganisms.

The effects of cannabinoids on the development and maintenance of infections in pre-clinical models are still not fully understood. According to the aforementioned findings, it appears that cannabinoids, particularly ∆^9^-THC (10), could weaken the immune system and render it ineffective towards intracellular pathogens [150]. However, cannabinoids may also be helpful in protecting against extracellular bacterial attacks and the damage induced by an overactive immune response in bacterial infections. Despite the progress that has been made in the field of bacterial targets and the development of new antimicrobial methods, more research needs to be conducted in order to understand the role of cannabinoids in the treatment of various infections. Concerns about the safety and toxicity of cannabis extract products have been greatly reduced by the use of non-psychotropic cannabinoids, which have shown in vitro properties that are capable of fighting against bacterial infections. According to all data in this study, cannabinoids and other cannabis constituents exhibited some impressive in vitro antibacterial properties that should be further explored in the search for novel substances that could potentially function as antimicrobial agents against clinically significant bacteria.

## 9. Conclusions and Outlook

*C. sativa* is a plant with an untapped potential. This versatile plant can be used for various purposes. Given its complex metabolic profile and excessive use as a recreational substance, its therapeutic benefits should not be ignored or overshadowed. Due to the limited effectiveness of antibiotics against MDR bacteria, the use of these drugs can be limited. This is why the discovery of an antimicrobial agent that can be used by plants has been regarded as a great step in the development of anti-infectives [8]. Multiple cannabinoids have been shown to have potent antimicrobial properties against Gram-positive pathogens, such as MRSA. In vitro studies have shown that cannabinoids can be useful in the removal of harmful microbes from the environment. Combination therapy with antibiotics that have different modes of action has shown broad-spectrum activities and synergism. There is also evidence that compounds found in *C. sativa* can have antimicrobial properties. This suggests that further investigations are needed to understand their potential. As the development of antibiotic resistance continues, cannabinoids have the potential to become a new source of treatment for bacterial infections.

Due to the complex pharmacology of cannabigerol (CBG) (17), it is not possible to determine the exact pharmacological properties of this substance in the endocannabinoid system [151,152,153]. This is because the role of this receptor in the development of various physiological processes, such as the brain and embryo, is very important. To fully comprehend the relationship between CBG (17) and the endocannabinoid system, more research is required. Although the activity of CBG (17) in human erythrocytes has been associated with a low therapeutic index, preliminary results show that the drug does not cause acute toxicity in both rats and mice [51,154]. Moreover, it is crucial to stress that cannabinoids have the potential to be addictive due to their capacity to stimulate the reward system, and prolonged usage may result in tolerance and dependency [155,156]. The short-term administration of antimicrobial drugs can lead to drug resistance. This is not a major concern since it would be very unlikely for bacteria to develop resistance in the body over long periods. Despite its various physical properties, such as its molecular weight, and number of acceptors and rings, CBG (17) is not considered a promising candidate due to its high lipophilicity. Its poor water solubility is also a major issue that needs to be resolved in the development of effective medicinal chemistry compounds. Due to the adipocytes’ incredibly lipophilic nature, additional research is necessary in order to rule out any adverse long-term effects associated with the accumulation of therapeutic cannabis in fatty tissues [157]. The ability to isolate and synthesis CBG (17) from high content sources such as *C. sativa* is beneficial for the development of effective antimicrobial agents [10,158]. This will allow one to explore various chemical properties of this plant.

## Figures and Tables

**Table 2:** Current pre-clinical in vitro research on different cannabinoids in cancer cell lines.

Cancer Cell Lines	Cannabinoid (s)	Inhibitory Concentrations	In Vitro Activity	Ref.
	WIN 55, JWH-133, AM251, SR144528	0–10 µM	Both CB_1_ and CB_2_ receptors are expressed by all cell lines. COX-2 signalling and apoptosis-mediated inhibition of cell migration and proliferation	[89]
	CBD (10), Capazepine, AM251, AM630	0–10 µM	Reduced cell viability, ER stress-induced autophagy and apoptosis, suppression of Akt, and mTOR signalling	[90]
Human breast adenocarcinoma	CBD (10)	1.5 µM	Inhibition of cell growth and invasion is achieved via modifying ERK and ROS, downregulating Id-1 expression, and upregulating Id-2 expression.	[91]
	AEA, AM251	0–0.5 µM	Decrease in the invasiveness of CD44^+^/CD24^−/low^/ESA^+^ cancer stem cell	[92]
	CBDA (11), ST-247, GSK0660, GW501516	1–50 µM	CBDA (11) prevents transcriptional activation of PPARβ/δ	[93]
	CBD (10)	1–50 µM	A synergistic effect observed after co-administration of CBD_sol_ and paclitaxel or docetaxel	[94]
Human glioblastoma	Δ^9^-THC (1), CBD (10)	0–5 µM	The substantial apoptotic induction and GIC population reduction	[95]
	CBD (10)	0–5 µM	Downregulation of key stem cell regulators including Sox2 and p-STAT3 and activation of p-p38 pathway	[96]
	CBD (10), SR141716, SR144528	5–40 µM	Effects on apoptosis induction and antiproliferative activity	[96]
Human neuroblastoma	Δ^9^-THC (1), CBD (10)	0–50 µg/mL	Cell viability reduction and apoptosis	[97]
Human glioblastoma multiforme, Human GBM cultures	Δ^9^-THC (1), WIN 55,212–2	0.1 nM–2 µM	Increase in apoptosis and antiproliferative effects	[98]
Pancreatic cancer	CBD (10)	0–10 µM	GPR55-mediated antiproliferative effects	[99]
Human colon cancer	SR141716	0–20 µM	Cell growth inhibition, a rise in caspase-3, and the cleavage of PARP	[100]
	SR141716	0.1–20 µM	Reduction in the growth of colon CSCs and tumour-derived cells	[101]
Human hepatocellular carcinoma	WIN 55, AM630, JWH-015	0, 5 or 10 µM	ERK1/2 phosphorylation is downregulated by CB_2_	[102]
Human gastric adenocarcinoma	AEA, Meth-AEA (R-(+)), CP 55,940	0.5–5 µM	Effects of concentrations on changes in cell morphology	[103]
	WIN 55, 212–2	5 µM	Prevention of cell invasion, migration, and EMT	[104]
Human prostate adenocarcinoma	AEA, 2-AG, Methanandamide (AM-356), SR141716	2.5, 5 and 10 µM	Induction of apoptosis and cell cycle arrest	[105]
	WIN 55, 212–2, SR141716, SR144528	0–10 µM	By inhibiting PI3K/Akt/mTOR signalling, WIN suppresses neuroendocrine differentiation	[106]
Human NSCLC; A549 (epithelial), CALU1 (mesenchymal)	JWH-015, SR144528	0–5 µM	Decreased ability to migrate and invade through reductions in FAK, VCAM1, and MMP2	[107]
Human lung cancer	WIN 55, 212–2	5–20 µM	Reduction in viability of cell due to apoptosis	[108]
Human myeloma	WIN 55, 212–2	5–50 µM	Apoptosis	[109]
Human T acute lymphoblastic leukaemia, Jurkat	CBD (10)	0.01–10 µM	Decreased in viability of cell and cell cycle arrest	[110]
Human melanoma	Δ^9^-THC (1), CBD (10)	0–10 µM	Decreased in viability of cell	[111]
Murine squamous, non-melanoma skin cancer	AEA, AMG9810, AM251, AM630	2.5–40 µM	Reduction in viability of cell due to apoptosis	[112]
Human renal carcinoma	WIN 55, 212–2, JWH-133, SR141716A, AM630	0–25 µM	Induction of apoptosis and reduction in cell proliferation	[113]
Human ovarian cancer	CBD (10)	10–50 µM	Inhibition of proliferation of cell	[94]
Rat adrenal gland	DHA-DA, AEA	0–80 µM	NOS activation, enhanced Ca^2+^ signalling, and GPR55 activation cause apoptosis	[114]

AEA (anandamide); MET-AEA (methanandamide, non-hydrolysable analogue of AEA); AM251 (CB_1_ antagonist); DHA-DA (N-docosahexaenoyl dopamine); HU-210 (CB_1_ agonist); JWH-133 (CB_2_ agonist); JWH-015 (CB_2_ agonist); SR141716 (CB_1_ inverse agonist); WIN 55,212–2 (CB_1_ agonist); N-oleoylethanolamine (NOE) (acidic ceramidase inhibitor); SR144528 (CB_2_ inverse agonist); PD98059 (ERK inhibitor); LY294002 (PI3K inhibitor); PBMCs (peripheral blood mononuclear cells); GW9662 (PPAR-γ antagonist); AM630 (CB_2_ antagonist); GSK066 (PPARβ/δ antagonist); AMG9810 (TRPV1 antagonist); GSK501516 (PPARδ antagonist); NOS (nitric oxide synthases); EMT (epithelial-mesenchymal transition); CSCs (cancer stem cells).

## Data Availability

Not applicable.
